# DWI as a Quantitative Biomarker in Predicting Chemotherapeutic Efficacy at Multitime Points on Gastric Cancer Lymph Nodes Metastases

**DOI:** 10.1097/MD.0000000000003236

**Published:** 2016-04-01

**Authors:** Jinman Zhong, Weiwei Zhao, Wanling Ma, Fang Ren, Shun Qi, Jianmin Zheng, Xifu Wang, Tianchu Lv, Zhanliang Su, Hong Yin, Jing Ren, Yi Huan

**Affiliations:** From the Department of Radiology (JZ, WZ, WM, FR, SQ, JZ, HY, JR, YH), Xijing Hospital, Fourth Military Medical University, Xi’an, Shaanxi, China; and Department of Radiology (XW, TL, ZS, JR), Feinberg School of Medicine, Northwestern University, Chicago, IL.

## Abstract

The purpose of the hypothesis testing is to determine that apparent diffusion coefficient (ADC) as an early biomarker can predict the metastatic lymph nodes’ (LNs) response to neoadjuvant chemotherapy in advanced gastric cancer (GC) in early stage.

From March 2011 to June 2015, 106 patients with advanced GC were enrolled in the study. Patients underwent conventional magnetic resonance imaging and functional diffusion weighted imaging before and 3 days, 7 days, 30 days, and 60 days following the standard chemotherapy. After surgery, among 3034 detected LNs, the positive group was divided into complete response (CR) group, partial response (PR) group, and stable disease (SD) group in accordance to the Response Evaluation Criteria in Solid Tumors (RECIST) 1.1. Mean ADCs, short/long diameters of LNs before chemotherapy between the whole positive and the negative LNs were compared by *t* test. Changes of mean ADCs in 3 groups were analyzed by 1-way ANOVA.

The mean ADC of the whole positive LNs was (1.145 ± 0.014) × 10^−3^ mm^2^/s, which was significantly lower than that of the whole negative LNs ([1.491 ± 0.010] × 10^−3^ mm^2^/s; *P* < 0.05). The means of both short/long diameters in the whole positive LNs were significantly longer than those in the whole negative LNs (*P* < 0.05). In CR, PR, and SD groups, the mean ADC of metastatic LNs on the 3rd day, 7th day, 13th day, and 16th day following the chemotherapy were all higher than that of LNs before chemotherapy, respectively (all *P* < 0.05). In addition, significant difference was found between mean ADCs in any 2 time points (all *P* < 0.05), except between mean ADCs in the 3rd day and in the 7th day of the chemotherapy.

In conclusion, ADC can be used as an early biomarker to predict the metastatic LNs’ response to neoadjuvant chemotherapy in advanced GC in early stage.

## INTRODUCTION

Gastric cancer (GC) is the 2nd leading cause of cancer-related deaths following lung cancer.^1–4^ Surgical resection is the pivotal curative treatment and is rely on the GC stage at presentation.^5–7^ Patients diagnosed with early-stage GC may mostly be cured with surgery alone and a 5-year survival rate is 70% to 95%.^8^ Unfortunately, most GC patients were diagnosed in advanced stage and the incidence of lymph nodes (LNs) involvement is extremely high.^9^ Surgery combined with neoadjuvant chemotherapy provides advanced patients much longer survival times than does initial surgery alone.^10^

Accurate radiological evaluation on chemotherapy response in early is indispensable to design appropriate therapeutic strategies for patients with advanced GC in time. In daily practice, conventional radiological imaging techniques such as computed tomography (CT) or morphological magnetic resonance imaging (MRI)^11,12^ are routinely used for follow-up of patients with GC, but they are insufficient to detect subtle changes and evaluate chemotherapy response in the earlier stage.

Diffusion weighted imaging (DWI) is a technique that measures the diffusion process of water within biological tissues noninvasively and has proven to be very sensitive for the detection of cell death including tumor apoptosis.^13–15^ DWI measurements of tumor tissue water mobility may be ideal for assessment of chemotherapy response in vivo. Moreover, as functional imaging technique, DWI has been demonstrated to detect early or subtle changes that cannot be found by conventional morphological MRI.

Recently, studies have showed DWI could help distinguish malignant lesions from benign ones,^11^ assess preoperative or postoperative tumor-node-metastasis staging accuracy,^16,17^ monitor the aggressiveness of GC,^18^ and assess response to treatment in local advanced GC.^19^ To the best of our knowledge, however, there have been no prior studies on DWI and apparent diffusion coefficient (ADC) values for assessing chemotherapeutic efficacy on metastatic LNs of advanced GC at multitime points.

In our study, we analyzed the changes of ADC values at different time points before and after chemotherapy in addition to the morphologic characteristics of LNs in conventional MRI, respectively, to investigate the following hypothesis that DWI as an early biomarker can predict the metastatic LNs’ response to neoadjuvant chemotherapy in advanced GC in early stage.

## MATERIALS AND METHODS

### Patient Population

**FIGURE 1:**
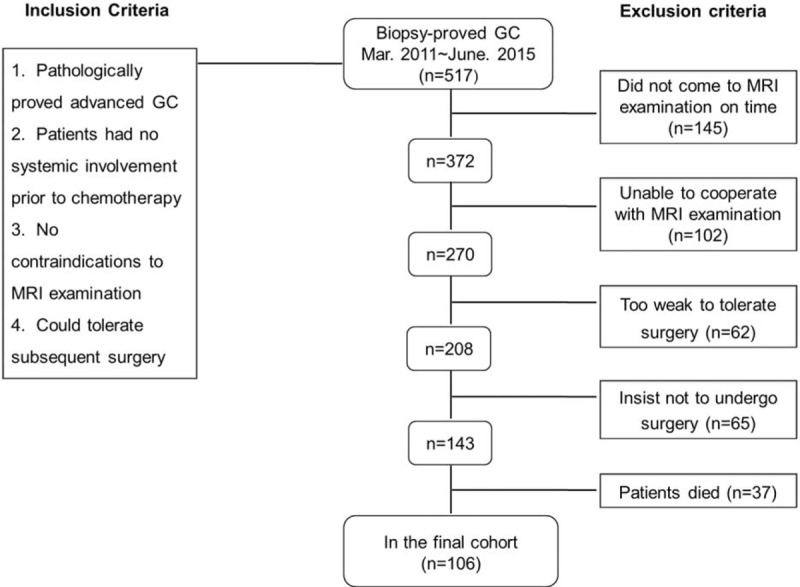
Flowchart showed the study enrollment. Of all the 571 patients, 106 were finally included. GC = gastric cancer.

This study was granted by the institutional review board of our hospital with a waiver for informed consent. From March 2011 to June 2015, 517 patients with biopsy-proved GC were referred from the Department of Gastroenterology in our hospital. For inclusion, the candidates should have documentation of eligibility criteria including: histologically confirmed diagnosis of primary GC by endoscopy; in the stage of advanced GC with discernible LNs (included any LN seen in images); had no systemic (stage IV) involvement via CT or MRI before chemotherapy; no contraindications to raceanisodamine hydrochloride injection and MRI examination; and could tolerate the surgery. Finally, among 517 patients, 411 were excluded due to various reasons. The case accrual process is summarized in Figure 1. In the final cohort, 106 patients were enrolled in this study (mean age 61 years; range 26–84 years; 47 male with a mean age of 54 years and age range of 26–79 years; 59 female with a mean age of 57 years and age range of 27–84 years).

### MRI Protocol

All patients were placed in the supine position and examined on a clinical 3.0 Tesla whole-body magnetic resonance system (Siemens Magnetom Trio, Erlangen, Germany) with an 18-channel surface phased-array body coil before chemotherapy and 3 days, 7 days, 30 days, and 60 days following the standard chemotherapy. Patients were asked to drink 1000 mL tap water half an hour before MR examination to enhance contrast between gastric wall and gastral cavity; 10 mg of intramuscular raceanisodamine hydrochloride injection was administered 10 min prior to the examinations to reduce the artifacts arising from peristaltic gastric movement.

The routine MRI and DWI protocols were showed in Table 1. After scanning, the image data were sent to the dedicated MRI (Leonardo; Siemens) image processing workstation.

**TABLE 1:**
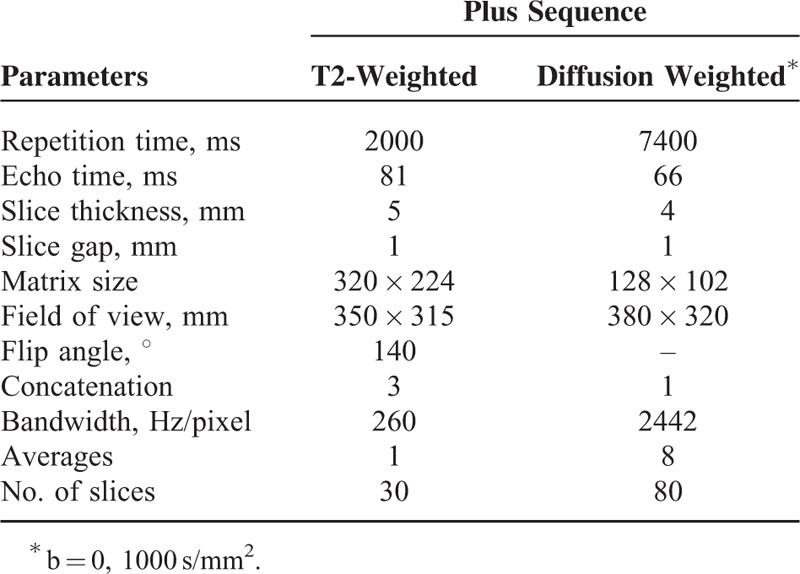
Magnetic Resonance Imaging Parameters

### Measurement of ADC Values and Short/Long Diameters of LNs

Two abdominal radiologists, each with more than 7 years experience in clinical MRI, were blinded to the final diagnosis and evaluated the acquired images independently. Another experienced radiologist with more than 20 years of experience reviewed the images and made the decision in consensus when the former 2 observers had differences in reading images.

The group, location, and signal intensity of each LN were identified and recorded on the conventional MRI (T2WI). With the reference of the T2WI, LNs were identified in the DWI. The ADC maps were automatically generated in the workstation (Siemens). In the largest slice of each LN, an oval-shaped region of interest (ROI), liquefaction and necrosis excluded, was marked on the DWI (b = 0 mm/s^2^) as a reference. The ROI was then pasted on the ADC map with b = 1000 mm/s^2^. For improving measurement precision, ADC measurements were averaged between the 2 observers as the final ADC value of each LN at multitime points.

In addition, the short/long diameter of each LN in the positive group and the negative group was also measured at different time points. The measurements of 2 observers were averaged as the final short/long diameter of the LN. The reduction of area of LNs was calculated via the formula followed: (short diameters of LNs × length to diameters of LNs before chemotherapy − short diameters of LNs × length to diameters of LNs 60 days after commencing chemotherapy)/short diameters of LNs × length to diameters of LNs before chemotherapy.

### Therapeutic Regimen

Patients were administrated chemotherapy of FOLFOX6 (Oxaliplatin [Hengrui Pharmaceutical Co., Ltd, Jiangsu, China], calcium folinate [Hengrui Pharmaceutical Co., Ltd], and 5-fluorouracil [Xudong Haipu Pharmaceutical Co., Ltd, Shanghai, China]). Sixty days after commencing chemotherapy, radical gastrectomy with extended lymphadenectomy was performed among the 106 patients. The extended lymphadenectomy procedure included total resection of LNs in metastatic N1 and N2 stations, during which perigastric nodes (N1 station) in addition to the LNs around the left gastric artery, the common hepatic artery, the coeliac axis, the splenic hilus, and the splenic artery (N2 station) were removed.^20,21^ Primary tumor was dissected.

### Histopathological Analysis and Grouping

The resected LNs were sent to the Department of Pathology in our hospital for further analysis. A total of 3034 LNs were marked and classified into 3 groups by the identified 16 LNs compartments surrounding stomach^22^ based on the guidelines of the Japanese Research Society for the Study of Gastric Cancer^20^: positive group (471 LNs, 51 compartments, LNs in each compartment were all metastatic based on the histopathology), negative group (1106 LNs, 103 compartments, LNs in each compartment were all nonmetastatic based on the histopathology), and partial positive group (1457 LNs, 223 compartments, in this group, there were not only metastatic but nonmetastatic LNs in each compartment based on the histopathology).

We chose the positive group and divided it into 4 subgroups according to Response Evaluation Criteria in Solid Tumors (RECIST) 1.1 guideline^23,24^:

1. Complete response (CR) group (167 LNs): LNs of GC in the group were all disappeared at images.
2. Partial response (PR) group (189 LNs): at least a 30% decrease in the sum of diameters of LNs at images.
3. Stable disease (SD) group (115 LNs): neither sufficient shrinkage to qualify for PR nor sufficient increase to qualify for progressive disease (PD) at images.
4. PD group (0 LNs): at least a 20% increase in the sum of diameters of LNs at images.

LNs were matched between MR images and pathological features through LNs compartment-by-LNs compartment matching. The nodal matching was ensured by the 2 radiologists and the gastrointestinal surgeons involved in the surgery.

### Statistical Analysis

The measurement data including ADC values and short/long diameters of LNs were given as the mean ± standard deviation. The intra-class correlation coefficient (ICC)^25,26^ was used to evaluate the interobserver agreement between the 2 radiologists for ADC values and short/long diameters’ measurements of LNs. The mean ADC values and short/long diameters of LNs before chemotherapy between the whole positive LNs and the whole negative LNs were compared by 2-sample *t* test. Mean ADC values of different time points were compared by 1-way ANOVA in CR, PR, and SD groups, respectively. Then the difference of mean ADC values between any 2 time points in the 3 groups and the difference of mean ADC values between the same time points in any 2 groups were compared, respectively, through pair-wise comparison of Newman–Kueuls *q* test. Significance tests were 2-sided and a *P* value of <0.05 was considered as statistical significance. Analyses were performed with SPSS statistical software (Version 20.0 for Windows; SPSS, Inc., Chicago, IL).

## RESULTS

### Interobserver Agreement in Imaging Analysis

The measurements of ADC values and short/long diameters had excellent interobserver reproducibility. Of all the LNs in the positive group and the negative group, the interobserver agreement showed an ICC of 0.874 (95% confidence interval [CI], 0.815–0.932) in the ADC values. In addition, the agreement between the 2 observers was obtained in the short/long diameters’ measurements with ICC of 0.853 (95% CI, 0.782–0.907) and 0.871 (95% CI, 0.814–0.915), respectively.

### Comparison of the Mean ADC Values and Diameters Between the Positive LNs and the Negative LNs Before Chemotherapy

**FIGURE 2:**
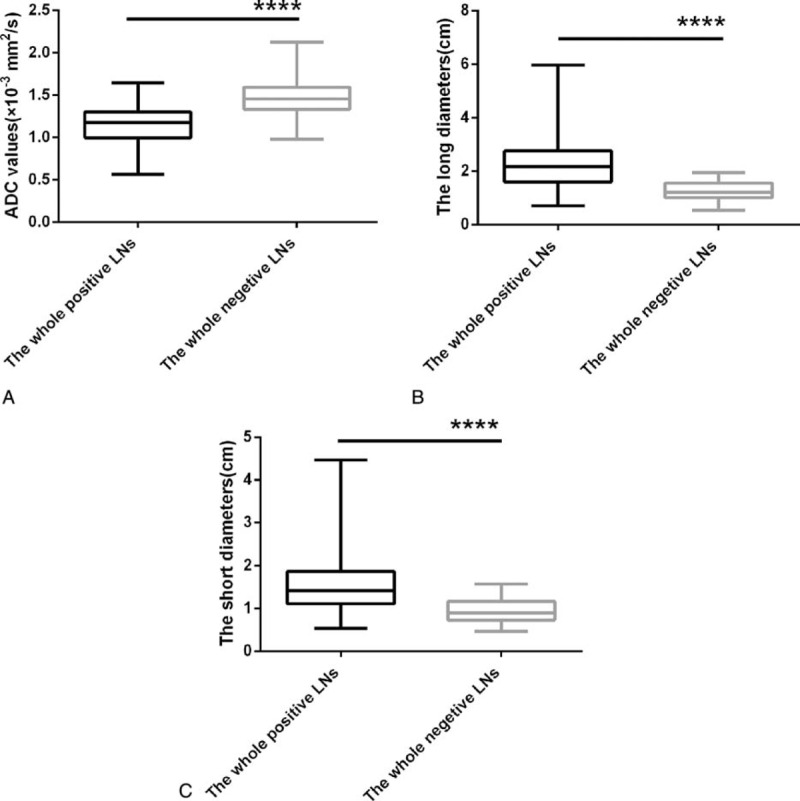
Box and whisker plot comparing the mean ADC value as well as the means of short/long diameter between the whole positive LNs and the whole negative LNs, respectively. The mean ADC value of the whole positive LNs was significantly lower than that of the whole negative LNs (A). The means of both short/long diameters in the whole positive LNs was significantly longer than those in the whole negative LNs, respectively (B, C). The middle line represents the median. The central box represents the measurements from the lower to the upper quartile (25–75 percentile). Whiskers indicate the range from the maximum to the minimum calculated the ADC and short/long diameter measurements. ADC = apparent diffusion coefficient, LNs = lymph nodes.

The mean ADC value of the whole positive LNs was (1.145 ± 0.014) × 10^−3^ mm^2^/s, which was significantly lower than that of the whole negative LNs (1.491 ± 0.010) × 10^−3^ mm^2^/s. The means of short diameters of the whole positive LNs and the whole negative LNs was 1.542 ± 0.041 and 0.939 ± 0.012 cm, respectively (*P* < 0.05). The mean long diameters of the whole positive LNs and the whole negative LNs were 2.314 ± 0.061 and 1.266 ± 0.014 cm, respectively (*P* < 0.05). Compared with the negative LNs, the positive LNs showed significantly longer mean diameter (Figure 2).

### Distribution of LNs in the Positive Group

The positive group contained 471 metastatic LNs which included 51 compartments in total cut from 47 patients. Each of the patients had a single known primary malignancy in the stomach. Among the tumors, 7 were in the gastric cardia, 7 in the gastric fundus, 13 in the gastric body, 4 in the gastric angle, 11 in sinus ventriculi, 3 in pylorus, and 2 was gastric infiltrating carcinoma, as were showed in Table 2. Of all the LNs in the positive group, 167 belonged to CR group, 189 to PR group, 115 to SD group, and 0 to PD group. In addition, the first 3 groups included 15, 29, and 7 compartments, respectively.

**TABLE 2:**
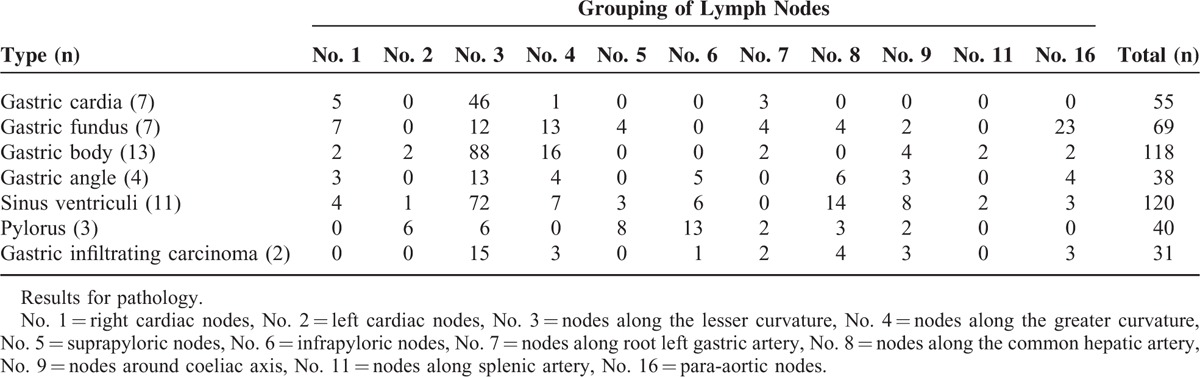
Distribution of Lymph Nodes in the Positive Group

### Imaging Characteristics of LNs in the Positive Group

**FIGURE 3:**
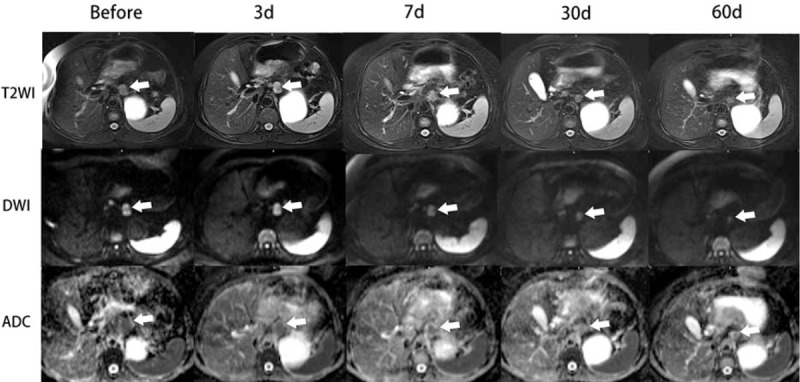
A 48-y-old woman with advanced GC and local metastatic LNs in PR group. Five images in each sequence (axial T2WI, DWI, and ADC maps) representing images before chemotherapy and 3 d, 7 d, 30 d, and 60 d after commencing chemotherapy, respectively. Axial T2WIs showing the high signal intensity of para-aortic LN (arrow) which was getting smaller and smaller among multitime points (images in the first row). On axial DWI at b = 1000 s/mm^2^, the LN showed high signal intensity which declined gradually as time went by (images in the 2nd row). ADC maps showing increased signal intensity of LNs simultaneously (images in the third row). ADC = apparent diffusion coefficient, DWI = diffusion weighted imaging, GC = gastric cancer, LNs = lymph nodes, PR = partial response.

LNs of the positive group demonstrated slightly hyperintense round-like nodes in T2WI, high signal intensity on DWI while relatively low signal intensity on ADC maps before chemotherapy. During the chemotherapy, the sizes of LNs decreased obviously in CR group, decreased modestly in PR group, and showed little change in SD group in T2WI; the signal intensity showed little change in T2WI, while declined gradually on DWI and increased on ADC maps simultaneously in these 3 groups. Figure 3 was obtained from a 48-year-old female patient with advanced GC, which illustrated imaging characteristics of the LNs in PR group before and after commencing chemotherapy.

### Quantitative Comparison of Measurements of ADC Values in the Positive Group

**FIGURE 4:**
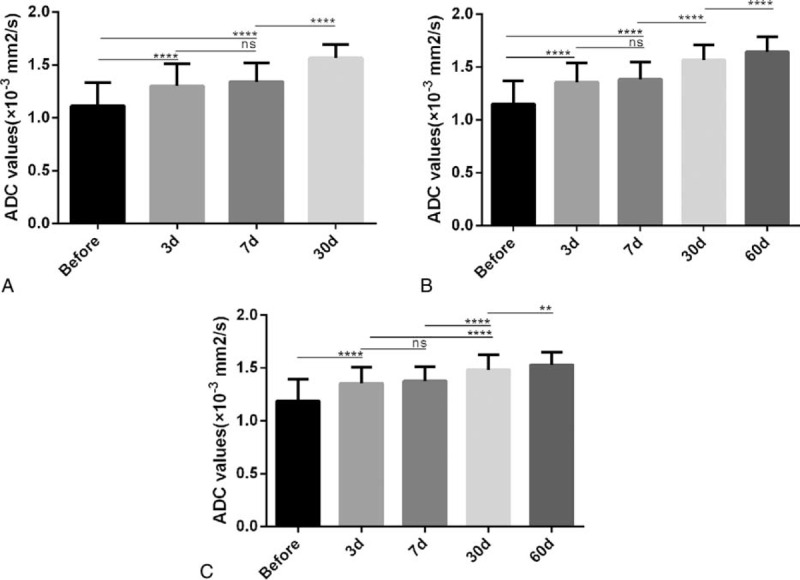
Vertical column bar graphs comparing the mean ADC values between any 2 time points before chemotherapy and 3 d, 7 d, 30 d, and 60 d after commencing chemotherapy in CR (A), PR (B), and SD (C) groups, respectively. The mean ADC values of metastatic LNs before chemotherapy were significantly lower than those of LNs after therapy across the measurement time points in the 3 groups. During the chemotherapy, there was significant statistical difference between the mean ADC values in any 2 time points, except the ADC values between the 3rd d and the 7th d in these groups. ADC = apparent diffusion coefficient, CR = complete response, LNs = lymph nodes, PR = partial response, SD = stable disease.

**FIGURE 5:**
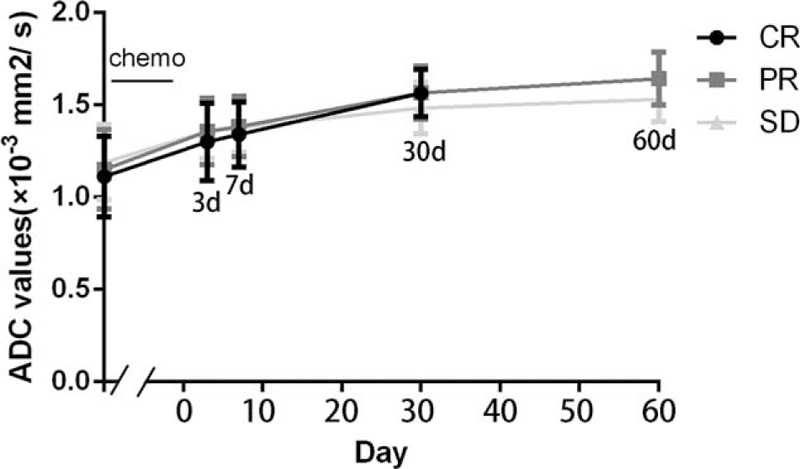
Line graph comparing the mean ADC values between any 2 same time points in the 3 groups. There was no statistical difference between any 2 same time points in any 2 groups. This graph also showed the variation trend of ADC values during the treatment of chemotherapy. The mean ADC values in CR, PR, and SD groups were all gradually on the rise across the measurement time points. ADC = apparent diffusion coefficient, CR = complete response, PR = partial response, SD = stable disease.

Four hundred seventy-one metastatic LNs had ADC values measurements recorded. Mean value and standard deviation of ADC values of LNs at multitime points in CR, PR, and SD groups were presented in Table 3. Some LNs in the 13th day and the whole LNs in the 16th day of chemotherapy in CR group cannot be found in images. Hence, we supplemented the lost ADC values of the 13th day of chemotherapy by mean/mode completer and did not count on ADC values of the 16th day of chemotherapy. Mean ADC values between any 2 time points in 3 groups were compared, respectively. Transform these statistical data into vertical column bar graphs, from which mean ADC values of metastatic LNs before chemotherapy were significantly lower than those of LNs after the initiation of the therapy across the measurement time points in 3 groups, respectively. Especially to deserve to be mentioned, the changes of mean ADC values became visible apparently as early as the 3rd day of chemotherapy. During the chemotherapy, there was significant difference between the mean ADC values in any 2 time points in 3 groups by pair-wise comparison, except between mean ADC values on the 3rd day and the 7th day in 3 groups (Table 4; Figure 4). Mean ADC values between 2 same time points in any 2 groups were compared as well, but no statistical difference was found. Furthermore, variation trends of mean ADC values in 3 groups were all gradually on the rise across the measurement time points (Figure 5).

**TABLE 3:**
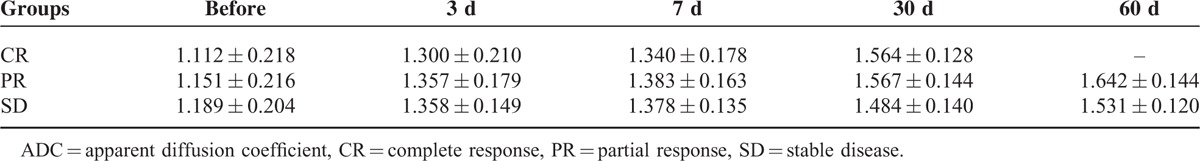
Mean ADC Values at Multitime Points in CR, PR, and SD Groups

**TABLE 4:**
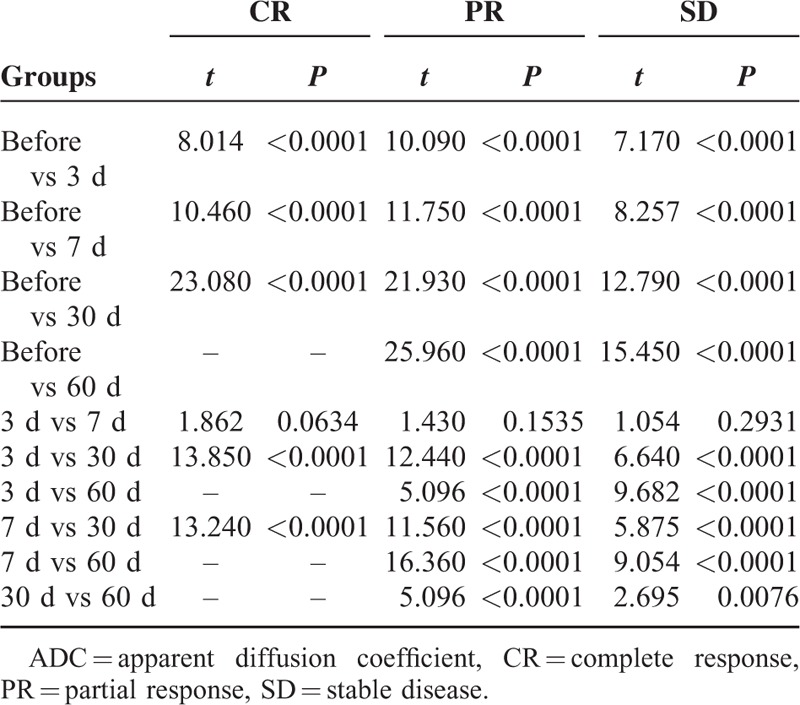
Comparison of Mean ADC Values Between Each 2 Time Points in CR, PR, and SD Groups

## DISCUSSION

The results of this study showed that ADC as an imaging biomarker can predict the metastatic LNs’ response to neoadjuvant chemotherapy in advanced GC in early stage. DWI has been used in evaluation of the malignancy via exploring the random motion of water molecules in vivo. The degree of restriction to water diffusion is partly related to the tissue cellularity and the integrity of cell membranes.^27–29^ The histopathological characteristics of malignancy are high cellular density correlated with numerous intact cell membranes. These features could diminish both the extracellular and intracellular spaces, and therefore cause a decrease in ADC values.^27,29^ In addition to the high cellular density, fibrosis and peritumoral nodal immunoreactivity probably have contributed to the restricted water diffusion.^30,31^ In contrast, ADC values of the benign lesions are usually on the high side exactly due to low cellularity compared with the former.^27^ Similarly, as for the lesions under or after appropriate treatment such as radiotherapy, chemotherapy or treatment in combination, the ADC values are on the rise. In addition, cell apoptosis and tissue necrosis caused by aggressive treatment promote the reduction of ADC values as well.^32^ In our study, the mean ADC value of the whole positive LNs was significantly lower than that of the whole negative LNs, which was consistent with the prior research results.^11,33–36^ The means of short/long diameters in the whole positive LNs was also significantly longer than those in the whole negative LNs, respectively. These findings are also consistent with previous investigations.^37^

Moreover, we analyzed changes of mean ADC values at different time points before and after commencing chemotherapy in addition to the morphologic characteristics of LNs in T2WI. The results showed mean ADC values of metastatic LNs before chemotherapy were significantly lower than those of LNs at any time points during the chemotherapy in CR, PR, and SD groups. During the chemotherapy, there was significant difference between the mean ADC values in any 2 different time points in 3 groups, but there was no statistical difference between the mean ADC values of the 3rd day and the 7th day in 3 groups, probably due to the short-time interval that was not enough to make ADC values to change obviously. Furthermore, the mean ADC values in 3 groups were all gradually on the rise across the measurement time points, but no statistical difference was found between the mean ADC values at any 2 same time points in any 2 groups, which was probably due to these 3 groups all in malignance. Although the sizes of LNs in CR and PR groups decreased with continuous treatment of chemotherapy, the changes of mean ADC values appeared much earlier (in the stage of the 3rd day after commencing chemotherapy) than morphological MRI (T2WI). Furthermore, several previous studies also demonstrated that the size of LNs is not a reliable predictive factor for evaluation of malignant LNs compared with DWI and ADC values.^38,39^ Therefore, we employed ADC values to predict the metastatic LNs’ response to neoadjuvant chemotherapy at an earlier stage over routine T2WI sequence along in GC.

In addition, LNs of GC are hard to trace in images corresponding with pathology simultaneously 1 by 1, due to the relatively small size but large number of draining LNs.^11,20^ As such, no prior studies have focused on assessing changes of ADC values of LNs with a multitime-point follow-up protocol in pathologic-radiologic correlation in GC. Hence, to solve this problem, our study performed, not a node-by-node comparison, but an LNs compartment-by-LNs compartment comparison between MR images and the pathology. We grouped the whole LNs based on the identified draining LNs compartments surrounding stomach in GC.^20^

This study possesses limitations. First, we analyzed these data by summary statistics, not by single datum, which may neglect variability and heterogeneity of each LN. A second limitation is that we classified LNs by identified draining LNs compartments surrounding stomach, which was not a consistent 1-to-1 match between images and pathology. Finally, DW images were obtained with only 2 b-values (0 and 1000 s/m^2^), although this is a frequently used strategy. In this sense, it is still not clear which are the optimal set of b-values and should be improved in the future studies.

In conclusion, DWI can serve as a more sensitive way to predict metastatic LNs’ response to chemotherapy compared with morphological MRI in GC. There is a promising benefit of this radiation-free technique for selecting the optimal therapeutic regimen for GC patients.
